# Comparative effects of final canal irrigation with chitosan and EDTA

**DOI:** 10.1590/1678-7757-2019-0005

**Published:** 2019-11-15

**Authors:** Polliana Vilaça Silva Antunes, Luis Eduardo Souza Flamini, Jardel Francisco Mazzi Chaves, Ricardo Gariba Silva, Antonio Miranda da Cruz

**Affiliations:** 1 Universidade de São Paulo, Faculdade de Odontologia de Ribeirão Preto, Departamento de Odontologia Restauradora, Ribeirão Preto, SP, Brasil.

**Keywords:** Chitosan, EDTA, Confocal microscopy

## Abstract

Chitosan is a natural, biocompatible chelating substance with potential for dental use. This study compared the effects of final canal irrigation with chitosan and EDTA on dentin microhardness, sealer dentin tubules penetration capacity, and push-out strength.

**Methodology:**

Fifty canine roots were distributed according to the final irrigation protocol (n=10): G1- 15% EDTA with conventional irrigation; G2- 15% EDTA with Endovac; G3- 0.2% chitosan with conventional irrigation; G4- 0.2% chitosan with Endovac; and G5- without irrigation. Specimens were obturated (AH Plus) and sectioned in 3 slices per root third. The first slice was used for microhardness and sealer penetration assessments under a laser confocal microscope. The second was utilized in a push-out strength test. The third slice was discarded. Data were analyzed using 2-way ANOVA and Tukey's *post hoc* test (α<0.05). Failure mode was determined at x40 magnification.

**Results:**

Microhardness reduction was more significant in groups G2 and G4 (p<0.05). Sealer penetration through dentin was significantly greater in group G2 (p<0.05). There was no significant difference between groups G1, G3, and G4 (p>0.05). In general, all experimental groups presented similar bond resistance (p>0.05) that significantly differed from the control (p<0.001). Mixed type failures were predominant.

**Conclusions:**

In general, 0.2% chitosan and 15% EDTA solutions act in a similar manner with regard to the variables studied. The use of Endovac potentiates the effect of these solutions.

## Introduction

The use of auxiliary chelating solutions for final irrigation of the root canal generally aims to promote the removal of the smear layer. This layer prevents intimate contact between the endodontic sealer and dentin walls, impairing sealer adhesion^1^ and consequently reducing the bond strength of the sealer mass.^2^ In particular, bond strength is considered an extremely relevant factor regarding filling quality.^3^

A dentine-free smear layer allows penetration of the sealer into the dentin tubule. In addition to favoring the sealer's mechanical retention,^4^ this phenomenon may be biologically beneficial, as sealers have an antibacterial effect on the infected dentin.^5^ A published report has recommended the removal of the smear layer through the irrigation of the root canal with sodium hypochlorite followed by a final irrigation with EDTA.^6^ Despite removing the smear layer, this association promotes dentin erosion^7^^,^^8^ and reduces microhardness.^9^ Biocompatible and less aggressive dental chelating solutions have therefore been proposed for this purpose.^10^^,^^11^ Chitosan, a natural polysaccharide, is prominently used in dentistry because it is biocompatible, biodegradable, bioadhesive, and non-toxic, with broad-spectrum antimicrobial properties and chelating activity.^12^^,^^13^ Chitosan has the ability to remove the smear layer and unblock dentin tubules without promoting significant dentin erosion.^10^ This substance has been proposed as an alternative solution to EDTA, which has toxic and pollutant effects.^14^ According to the literature, final irrigation of the root canal with chitosan has the advantage of removing the smear layer in addition to inhibiting bacterial recolonization.^15^

The irrigation of the root canal with a negative pressure system has been reported to be more efficient than the conventional system, contributing to improve the cleaning of dentin walls.^16^ This favors the penetration of the endodontic sealer into the dentin tubule enabling antibacterial activity on the infected dentin.^17^ Resin sealers, compared with zinc oxide-based sealers, have been reported to have greater dentin tubule penetration ability and can adhere to the dentin walls.^18^

Accordingly, the present study compared the effects of final canal irrigation with chitosan and EDTA, with and without the use of a negative pressure system, on dentin microhardness, sealer *canaliculi* penetration capacity, and bond resistance. The null hypothesis was that, regardless of the irrigation system applied, chelating solutions have similar effects in relation to dentin microhardness, sealer *canaliculi* penetration capacity, and bond resistance.

## Methodology

After the approval by the Research Ethics Committee (No.: 16219413.7.0000.5419) fifty human canines extracted due to periodontal problems from patients ranging in age from 45 to 60 years were selected. The teeth were stored in 0.1% thymol solution at 9°C until the experiments, when they were washed in water for 24 hours. The outer root surface of each tooth was cleaned by scraping it with ultrasound. Subsequently, the teeth were evaluated visually and radiographically in order to select healthy specimens with fully-formed root and single-canals. The dental crown was removed so that the remaining roots were 21 mm long. The working length of each root canal was determined by subtracting 1 mm from the length where size 10 was visible at the apical foramen. After the impermeabilization of the entire root portion with cyanoacrylate, a biomechanical preparation was performed with instrument #R50 from the Reciproc^®^ system (VDW, Munich, Germany) and NaOCl 1%, according to the manufacturer's recommendation. During preparation, 2 mL of a 1% sodium hypochlorite solution was used after three pecking motions. After drying them with an absorbent paper cone, the roots were distributed between 5 groups (n=10) according to the final irrigation protocol: G1- conventional irrigation with 5 mL of 15% EDTA; G2- irrigation with Endovac with 5 mL of 15% EDTA; G3- conventional irrigation with 5 mL of 0.2% chitosan solution; G4- irrigation with Endovac with 5 mL of 0.2% chitosan; and G5- control group (without final irrigation). Final irrigation in groups G1 and G3 were performed using a Luer lock syringe connected to a NaviTip plastic cannula. In groups G2 and G4, irrigation occurred through the EndoVac system (Discus Dental, Culver City, CA, USA). In all experimental groups, irrigation of the chelating solution was performed with a flow rate of 5 mL for 3 minutes. After irrigation, the canal received 20 mL of distilled water.

The canals were dried and obturated with #R50 gutta-percha cones and supplementary gutta-percha cones and AH Plus resin sealer (Dentsply, DeTrey, Konstanz, Germany). The samples were kept at 100% relative humidity and 37°C for 72 hours. The roots were cut transversely, obtaining 3 slices *per* third with a thickness of 2±0.2 mm. The first slice of each third was used in microhardness and laser confocal microscopy analyses, while the second was utilized in the push-out test and the third was discarded.

### Analysis of dentin microhardness

The cervical surface of the first slice was sanded with 500, 600, and 1,200 grit water sandpaper for 2 minutes in each grit, followed by polishing with a felt disc (Diamond, FGM, São Paulo, Brazil) coupled to a polisher (Panambra Struers DP-10-Panambra, São Paulo, Brazil), with the aid of alumina paste (Arotec, Cotia, São Paulo, Brazil). The specimens were placed in ultrasonic water-container vats for 3 minutes to remove the paste residue. The smoothness of the surface was verified by a magnifying glass (x40). A Knoop HMV-2 hardness apparatus (Shimadzu HMV-2000, Shimadzu Corporation, Kyoto, Japan) was used to measure the microhardness with a load of 10 g for 15 seconds. Five indentations were made in each slice, starting from the root canal lumen towards the cement, with distances of 100 μm between them.

### Laser confocal microscopy analysis

**Figure 1:**
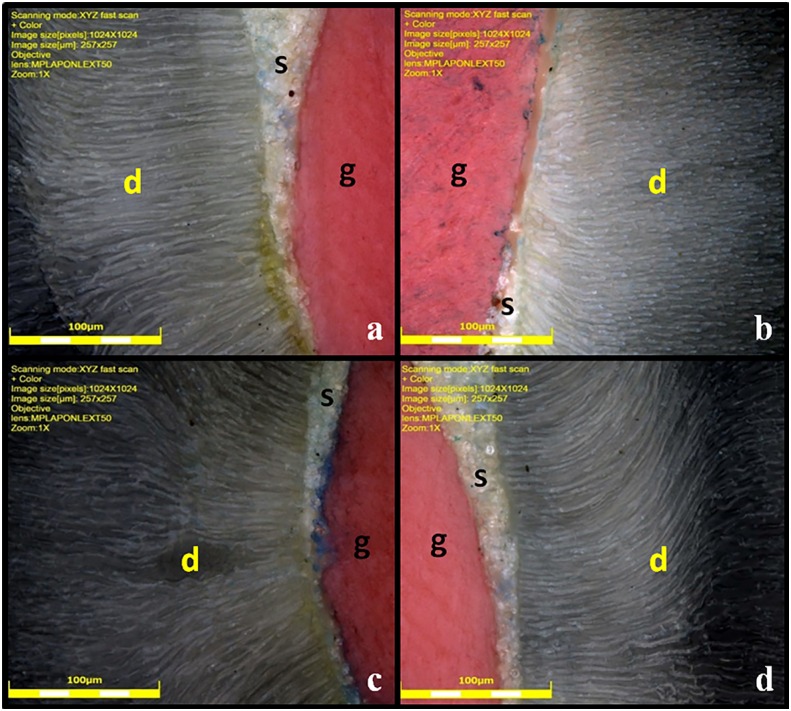
Confocal laser microscopy images (50×) at upper left (a), upper right (b), lower left (c), and lower right (d) quadrant illustrate the endodontic sealer penetrating the dentinal tubules. g= gutta-percha; s= sealer; d= dentin

The cervical face of the slice was treated with 10% phosphoric acid, washed with distilled water, and then viewed under a confocal laser microscope (Lext OLS 4000, Olympus, Waltham, USA) to obtain images of the dentin/sealer interface. Subsequently, the depth at which the sealer penetrated the dentin tubules was measured. This measurement was performed in 4 quadrants: upper left (a), upper right (b), lower left (c), and lower right (d) (Figure 1). The measurement started from the wall of the root canal, following the trajectory of the dentinal *canaliculus* to the maximum point of penetration of the endodontic sealer. The specimens were analyzed with 50× magnification, 257x257 µm field of vision with 1024X1024 pixel resolution.

### Evaluation of the bond strength (push-out)

Shear strength by extrusion was evaluated by an Instron Universal Machine Model 3345 (Instron Corporation, Canton, MA, USA). The Instron machine was operated with constant application of compressive load at a crosshead speed of 0.5 mm/min until the sealer displacement. The force exerted for the displacement was measured in kiloNewton. To calculate the bond strength, the acquired force was transformed into Newtons and converted into Megapascals (MPA), and then divided by the lateral area of the sealer. The calculation of the lateral area was performed using the lateral cone area formula:

$$S_{L}=\text{π(R}+\text{r)}\sqrt{h^{2}{\text{(R}-\text{r)}}^{2}}$$

In this formula, “S_L_” corresponds to the lateral cone area, “R” to the radius of the sealer (coronal portion), “r” to the radius of the filling material (apical portion), and “h” to the height/thickness of the filling material.

### Type of failure analysis

After the measurement of the bond strength, both faces of the specimens were examined under an optical microscope (x40) to classify adhesion failure. Failure was considered adhesive if the sealer was totally separated from the dentin, cohesive if the fracture occurred within the sealer, and mixed when a combination of adhesive and cohesive modes occurred.

### Statistical analysis

The influence of intergroup and intragroup irrigation protocols on dentin microhardness, penetration of sealer, and push-out strength in the root regions (cervical, middle, and apical thirds) were evaluated by variance analysis (2-way ANOVA). The Tukey-Kramer complementary test (α=0.05) was applied for multiple comparisons. The level of significance was 5% (α<0.05). Statistical evaluation of adhesive failure comprised the percentage distribution of the types of failure found.

## Results

The intergroup and intragroup comparisons of means and standard deviation of dentin microhardness, sealer penetration through dentin tubules, and bond strength are presented in Tables 1, 2, and 3, respectively.

**Table 1:** Intergroup and intragroup comparisons regarding the means with standard deviation (SD) of the values of dentin microhardness (Knoop)

	G1	G2	G3	G4	G5
	Means ± SD	Means ± SD	Means ± SD	Means ± SD	Means ± SD
Cervical Third	56.88±6.64^c,B^	52.71±6.77^b,C^	55.24±7.36^c,D^	50.31±5.64^a,E^	68.91±8.32^d,A^
Middle Third	48.53±6.55^a,A^	46.26±4.88^b,B^	45.41±5.13^b,B^	44.99±5.12^b,A^	67.28±6.52^c,A^
Apical Third	44.56±5.36^b,C^	42.54±3.21^b,B^	42.68±3.85^b,B^	42.61±3.09^b,B^	58.06±6.21^c,A^

* Different lower case letters in the lines mean significant intergroup statistical difference

* Different capital letters in the columns mean significant intragroup statistical difference

**Table 2:** Intergroup and intragroup comparisons regarding the means with standard deviation (SD) of the values (µm) of endodontic sealer penetration

	Cervical Third	Middle Third	Apical Third
	Means ± SD	Means ± SD	Means ± SD
G1	210.09^a,B^ ± 16.80	201.55^a,B^ ± 34.70	123.79^b,B^ ± 9.30
G2	212.92^a,A^ ± 37.81	247.62^a,A^ ± 36.06	88.52^b,B^ ± 5.32
G3	204.29^a,B^ ± 13.33	201.17^a,B^ ± 33.97	134.38^b,A^ ± 42.15
G4	159.55^a,B^ ± 48.05	165.80^a,B^ ± 77.65	141.63^a,A^ ± 10.95
G5	88.28^a,C^ ± 0.92	71.80^a,C^ ± 4.05	76.00^a,C^ ± 4.13

* Different lower case letters in the lines mean significant intragroup statistical difference in the thirds

* Different capital letters in the columns mean significant intergroup statistical difference in the same third

**Table 3:** Intergroup and intragroup comparisons regarding the means with standard deviation (SD) of the values of PS (MPA)

	Groups				
	G1	G2	G3	G4	G5
	Means ± SD	Means ± SD	Means ± SD	Means ± SD	Means ± SD
Cervical Third	2.91±0.59^b,A^	3.11±1.25^b,A^	3.08±1.20^b,A^	4.11±0.99^a,A^	0.76±0.68^c,A^
Middle Third	1.02±0.57^b,B^	2.34±0.88^a,B^	1.89±0.69^b,B^	2.90±0.94^a,B^	0.53±0.44^c,A^
Apical Third	0.93±0.19^a,B^	0.86±0.40^a,B^	1.04±0.41^a,B^	0.94±0.83^a,C^	0.30±0.12^b,A^

* Different lower case letters in the lines mean significant intergroup statistical difference in the same third

* Different capital letters in the columns mean significant intragroup statistical difference in the different thirds

### Dentin microhardness

In all treatment groups, dentin microhardness of the three root thirds was significantly reduced, as compared to that in G5 (p=0.001); microhardness reduction was more significant in G2 and G4 (p<0.05) (Table 1).

### Sealer penetration through dentin tubules

Greater sealer penetration was achieved in G2 than in the other groups (G2, G3, and G4) (p <0.05); while there were no differences between G2, G3, and G4, significant differences in the values of these groups as compared to that of G5 were observed (p> 0.05). In the intragroup comparisons between G1, G2, and G3, penetration depth of endodontic sealer at the cervical third was greater than at the apical third (p<0.005) (Table 2).

### Bond strength (push-out)

In intergroup comparisons of the cervical and apical thirds, similar values were achieved in G1, G2, G3, and G4 which had statistical difference as compared to those in G5 (p <0.001); in general, intragroup comparison revealed that the bond strength in the cervical third was significantly higher than those in the other thirds (p <0.001) (Table 3).

### Fracture pattern analysis

The types of failures recorded after shear stress are displayed in Table 4. In general, the percentage distribution of failure types observed showed predominance of cohesive failures in the cervical third, and a higher percentage of mixed type failures in the apical and middle thirds (Table 4).

**Table 4:** Distribution of failure modes (%) after the push-out test

	G1			G2			G3			G4			G5		
Failure	C	M	A	C	M	A	C	M	A	C	M	A	C	M	A
Adhesive	0	0	20	0	0	10	0	0	20	0	0	10	0	30	50
Mixed	30	70	70	20	80	80	20	60	80	20	70	80	40	40	30
Cohesive	70	30	10	80	20	10	80	40	0	80	30	10	60	30	20

* C, cervical third; M, middle third; A, apical third

## Discussion

This study compared the effects of final root canal irrigation with chitosan 0.2% and EDTA 15%, with conventional irrigation and the use of negative pressure irrigation, on dentin microhardness, sealer dentin tubules penetration capacity, and bond strength. The null hypothesis was partially confirmed because, although the solutions presented similar effects on microhardness and bond strength, there was a greater penetration of the endodontic sealer in the EDTA 15% group irrigated with EndoVac.

All groups showed significantly reduced dentin microhardness compared with the control group. This was expected since both EDTA and chitosan solutions are potent chelating agents.^9^ The most significant microhardness reduction occurred in the apical third of the canal. This is probably due to the greater number of dentinal *canaliculi* in the cervical portion, which indicates a greater amount of pericanalicular dentin, which in turn, is harder than intercanalicular dentin.^19^ The reduction in microhardness in the cervical and middle thirds was more pronounced in the groups irrigated with Endovac. Our findings agree with previous studies reporting that irrigation with a negative pressure system is more efficient than conventional irrigation, especially in the apical third.^20^^,^^21^ The literature does not report whether the chelating effect of the solution in the final irrigation significantly impacts the root fracture resistance. However, it is known that the reduction of dentin microhardness greatly facilitates the action of endodontic instruments during biomechanics. The decision of using a 0.2% chitosan solution compared with 15% EDTA was based on the study by Silva, et al.^10^ (2012), which evaluated the effect of different concentrations of chitosan on the dentin surface and smear layer removal. The authors verified that 0.1% chitosan used for 3 minutes removes the smear layer but not the smear plug. Chitosan at 0.2% used for the same amount of time showed visible dentin, open tubules, and slight erosion of peritubular dentin. Chitosan at 0.37% cleaned the dentin walls similarly to 0.2% chitosan, but with a much greater erosive effect. The present study did not compare the degree of erosion caused between 0.2% chitosan and 15% EDTA. However, given the similar effect between chitosan and EDTA on microhardness, one can state that, for this purpose, less concentrated solutions are preferable. It should be noted that chitosan is biocompatible, biodegradable, and non-toxic,^22^^,^^23^ in addition to possessing antibacterial activity.^15^

Unlike scanning electron microscopy, confocal microscopy does not require vacuum or metallization, which is responsible for sample dehydration and the occurrence of technical artifacts.^24^ Confocal microscopy has been widely used to observe and evaluate sealer penetration within the dentinal tubules.^25^ In the present study, EDTA irrigation with Endovac significantly favored sealer penetration compared with other groups. In order for the sealer to penetrate, dentin must be free of a smear layer and a smear plug.^26^ Therefore, the results suggest that EDTA used with negative pressure irrigation has a greater ability to remove these layers than chitosan. In contrast, a previous study reported that 0.2% chitosan removes the smear layer and unblocks dentinal tubules in a manner similar to EDTA.^27^ These contrasting results are probably due to the different irrigation systems used, conventional versus Endovac. We also observed that the penetration of the endodontic sealer was greater in the cervical and middle thirds. Camilleri^28^ (2015) reported that AH Plus penetrated the dentinal tubules of the coronal and middle thirds of the root, whereas in the apical third, penetration was not always observed. In addition to a greater number of dentinal tubules in this region, the *canaliculi* have larger diameters than the apical region.^29^ Clinically, the presence of the endodontic sealer inside the dentinal tubules allows the mechanical retention of the filling material^4^ and acts as a “blocking agent”, making bacterial repopulation difficult.^29^

The push-out test between groups showed that the bond strength observed in the third cervical was similar for both EDTA and chitosan groups, regardless of the irrigation system. The same was observed when comparing bond strength in the apical third. However, the analysis between the thirds showed that the bond strength in the cervical third was greater than in the middle and apical third. One explanation for this finding is the difficulty of the endodontic sealer to flow up to the apical third, in addition to the anatomical characteristic of dentin for each third in relation to the variation in the number and diameter of the dentinal tubules.^30^ Our findings are in concordance with previous studies.^31^^,^^32^ We observed that the increased bond strength in the cervical third is not necessarily related to greater sealer penetration in this third. This observation reinforces the assertion that there is no relationship between the bond strength and the depth of sealer tubule penetration.^33^^,^^34^

The fracture analysis of the slices showed that the predominant failures in the cervical third were cohesive, and mixed in the apical and middle thirds. These types of failures observed may be associated with the resin sealer used. Reports have shown that the AH Plus sealer presents a higher prevalence of mixed failures.^31^

Human teeth were used in this study. However, information of whether the teeth were necrotic or not at the time of extraction was not available, this represents a limitation. The use of necrotic teeth might potentially contain less tissue debris or contain variable amounts of bacterial biofilm.

The results obtained in the present study indicate that chitosan has the potential to be utilized as an alternative solution to EDTA in final root canal irrigation. However, it should be considered that prior to clinical use, further studies are necessary, especially in the biological research field, to assess the activity of this solution in humans.

## Conclusion

Considering the limitations of *in vitro* study, final irrigation of the root canal with 15% EDTA or 0.2% chitosan achieved comparable effects in terms of reducing dentin microhardness, penetrating endodontic sealer through the dentinal tubules, and bond strength. Endovac usage potentiated the effects of these chelators compared to that of conventional irrigation.

## References

[1] 1- Economides N, Liolios E, Kolokuris I, Beltes P. Long-term evaluation of the influence of smear layer removal on the sealing ability of different sealers. J Endod. 1999;25(2):123-5. 10.1016/S0099-2399(99)80010-7 10204470

[2] 2- Sagsen B, Ustün Y, Demirbuga S, Pala K. Push-out bond strength of two new calcium silicate-based endodontic sealers to root canal dentine. Int Endod J. 2011;44(12):1088-91. 10.1111/j.1365-2591.2011.01925.x 21895700

[3] 3- Souza MA, Rauber MG, Zuchi N, Bonacina LV, Ricci R, Dias CT, et al. Influence of final irrigation protocols and endodontic sealer on bond strength of root filling material with root dentin previously treated with photodynamic therapy. Photodiagnosis Photodyn Ther. 2019. doi: 10.1016/j.pdpdt.2019.03.013. 10.1016/j.pdpdt.2019.03.013 30902793

[4] 4- Ordinola-Zapata R, Bramante CM, Graeff MS, del Carpio Perochena A, Vivan RR, Camargo EJ, et al. Depth and percentage of penetration of endodontic sealers into dentinal tubules after root canal obturation using a lateral compaction technique: a confocal laser scanning microscopy study. Oral Surg Oral Med Oral Pathol Oral Radiol Endod. 2009;108(3):450-7. 10.1016/j.tripleo.2009.04.024 19716510

[5] 5- Arora S, Mir S, Gautam A, Batra R, Soni S, Lata K. Evaluation of antimicrobial efficacy of root canal sealers against *Enterococcus faecalis*: a comparative study. J Contemp Dent Pract. 2018;19(6):680-3. 29959296

[6] 6- Johal S, Baumgartner JC, Marshall JG. Comparison of the antimicrobial efficacy of 1.3% NaOCl/BioPure MTAD to 5.25% NaOCl/15% EDTA for root canal irrigation. J Endod. 2007;33(1):48-51. 10.1016/j.joen.2006.08.007 17185130

[7] 7- Qian W, Shen Y, Haapasalo M. Quantitative analysis of the effect of irrigant solution sequences on dentin erosion. J Endod. 2011;37(10):1437-41. 10.1016/j.joen.2011.06.005 21924198

[8] 8- Demirel A, Yüksel BN, Ziya M, Gümüş H, Doĝan S, Sari Ş. The effect of different irrigation protocols on smear layer removal in root canals of primary teeth: a SEM study. Acta Odontol Scand. 2019;77(5):380-5. 10.1080/00016357.2019.1577491 30859897

[9] 9- Cruz-Filho AM, Sousa-Neto MD, Savioli RN, Silva RG, Vansan LP, Pécora JD. Effect of chelating solutions on the microhardness of root canal lumen dentin. J Endod. 2011;37(3):358-62. 10.1016/j.joen.2010.12.001 21329821

[10] 10- Silva PV, Guedes DF, Pécora JD, Cruz-Filho AM. Time-dependent effects of chitosan on dentin structures. Braz Dent J. 2012;23(4):357-61. 10.1590/s0103-64402012000400008 23207849

[11] 11- Tuncel B, Nagas E, Cehreli Z, Uyanik O, Vallittu P, Lassila L. Effect of endodontic chelating solutions on the bond strength of endodontic sealers. Braz Oral Res. 2015;29. doi: 10.1590/1807-3107BOR-2015.vol29.0059. 10.1590/1807-3107BOR-2015.vol29.0059 25992788

[12] 12- DaSilva L, Finer Y, Friedman S, Basrani B, Kishen A. Biofilm formation within the interface of bovine root dentine treated with conjugated chitosan and sealer containing chitosan nanoparticles. J Endod. 2013;39(2):249-53. 10.1016/j.joen.2012.11.008 PMC3551251 23321239

[13] 13- Roshdy NN, Kataia EM, Helmy NA. Assessment of antibacterial activity of 2.5% NaOCl, Chitosan nano-particles against *Enterococcus faecalis* contaminating root canals with and without diode laser irradiation: an *in vitro* study. Acta Odontol Scand. 2018;1-5. doi: 10.1080/00016357.2018.1498125. 10.1080/00016357.2018.1498125 30152712

[14] 14- Spanó JC, Silva RG, Guedes DF, Sousa-Neto MD, Estrela C, Pécora JD. Atomic absorption spectrometry and scanning electron microscopy evaluation of concentration of calcium ions and smear layer removal with root canal chelators. J Endod. 2009;35(5):727-30. 10.1016/j.joen.2009.02.008 19410093

[15] 15- Del Carpio-Perochena A, Bramante CM, Duarte MA, Moura MR, Aouada FA, Kishen A. Chelating and antibacterial properties of chitosan nanoparticles on dentin. Restor Dent Endod. 2015;40(3):195-201. 10.5395/rde.2015.40.3.195 PMC4534723 26295022

[16] 16- Kumar VR, Bahuguna N, Manan R. Comparison of efficacy of various root canal irrigation systems in removal of smear layer generated at apical third: an SEM study. J Conserv Dent. 2015;18(3):252-6. 10.4103/0972-0707.157267 PMC4450535 26069415

[17] 17- Saleh IM, Ruyter IE, Haapasalo M, Ørstavik D. Survival of *Enterococcus faecalis* in infected dentinal tubules after root canal filling with different root canal sealers *in vitro*. Int Endod J. 2004;37(3):193-8. 10.1111/j.0143-2885.2004.00785.x 15009409

[18] 18- Sousa-Neto MD, Marchesan MA, Pécora JD, Junior AB, Silva-Sousa, YT, Saquy PC. Effect of Er:YAG laser on adhesion of root canal sealers. J Endod. 2002;28(3):185-7. 10.1097/00004770-200203000-00010 12017177

[19] 19- Luukko K, Kettunen P, Fristad I, Berggreen E. Estrutura e funções do complexo dentino-pulpar. In: Cohen S, Hargreaves KM. Caminhos da polpa. Elsevier: São Paulo; 2011. p. 418-63.

[20] 20- Kumar VR, Bahuguna N, Manan R. Comparison of efficacy of various root canal irrigation systems in removal of smear layer generated at apical third: an SEM study. J Conserv Dent. 2015;18(3):252-6. 10.4103/0972-0707.157267 PMC4450535 26069415

[21] 21- Widjiastuti I, Rudyanto D, Yuanita T, Bramantoro T, Aries Widodo W. Cleaning efficacy of root canal irrigation with positive and negative pressure system. Iran Endod J. 2018;13(3):398-402. 10.22037/iej.v13i3.20875 PMC6064011 30083214

[22] 22- Senel S, Kas HS, Squier CA. Application of chitosan in dental drug delivery and therapy. In: Muzzarelli RA, ed. Chitosan peros: from dietary supplement to drug carrier. Italy: Atec; 2000. p. 241-56.

[23] 23- Akncbay H, Senel S, Ay ZY. Application of chitosan gel in the treatment of chronic periodontitis. J Biomed Mater Res B Appl Biomater. 2007;80(2):290-6. 10.1002/jbm.b.30596 16767723

[24] 24- De-Deus G, Reis C, Di Giorgi K, Brandão MC, Audi C, Fidel RA. Interfacial adaptation of the Epiphany self-adhesive sealer to root dentin. Oral Surg Oral Med Oral Pathol Oral Radiol Endod. 2011;111(3):381-6. 10.1016/j.tripleo.2010.08.020 21169038

[25] 25- El Hachem R, Khalil I, Le Brun G, Pellen F, Le Jeune B, Daou M, et al. Dentinal tubule penetration of AH Plus, BC Sealer and a novel tricalcium silicate sealer: a confocal laser scanning microscopy study. Clin Oral Invest. 2019;23(4):1871-6. 10.1007/s00784-018-2632-6 30225679

[26] 26- Moon YM, Shon WJ, Baek SH, Bae KS, Kum KY, Lee W. Effect of final irrigation regimen on sealer penetration in curved root canals. J Endod. 2010;36(4):732-6. 10.1016/j.joen.2009.12.006 20307754

[27] 27- Silva PV, Guedes DF, Nakadi FV, Pécora JD, Cruz-Filho AM. Chitosan: a new solution for removal of smear layer after root canal instrumentation. Int Endod J. 2013;46(4):332-8. 10.1111/j.1365-2591.2012.02119.x 22970844

[28] 28- Camilleri J. Sealers and warm gutta-percha obturation techniques. J Endod. 2015;41(1):72-8. 10.1016/j.joen.2014.06.007 25115660

[29] 29- Amoroso-Silva PA, Guimarães BM, Marciano MA, Duarte MA, Cavenago BC, Ordinola-Zapata R, et al. Microscopic analysis of the quality of obturation and physical properties of MTA Fillapex. Microsc Res Tech. 2014;77(12):1031-6. 10.1002/jemt.22432 25209870

[30] 30- Tao L, Pashley DH. Shear bond strengths to dentin: effects of surface treatments, depth and position. Dent Mater. 1988;4(6):371-8. 10.1016/S0109-5641(88)80052-6 2978752

[31] 31- Carneiro SM, Sousa-Neto MD, Rached FA Jr, Miranda CE, Silva SR, Silva-Sousa YT. Push-out strength of root fillings with or without thermomechanical compaction. Int Endod J. 2012;45(9):821-8. 10.1111/j.1365-2591.2012.02039.x 22458910

[32] 32- Suzuki TY, Pereira MA, Gomes-Filho JE, Wang L, Assunção WG, Santos PH. Do irrigantion solutions influence the bond interface between glass fiber posts and dentin? Braz Dent J. 2019;30(2):106-16. 10.1590/0103-6440201901963 30970052

[33] 33- Machado R, Silva UX Neto, Carneiro E, Fariniuk LF, Westphalen VP, Cunha RS. Lack of correlation between tubular dentine cement penetration, adhesiveness and leakage in roots filled with gutta percha and an endodontic cement based on epoxy amine resin. J Appl Oral Sci. 2014;22(1):22-8. 10.1590/1678-775720130247 PMC3908761 24626245

[34] 34- Moinzadeh AT, Mirmohammadi H, Hensbergen IA, Wesselink PR, Shemesh H. The correlation between fluid transport and push-out strength in root canals filled with a methacrylate-based filling material. Int Endod J. 2015;48(2):193-8 10.1111/iej.12300 24749656

